# NP-59 Adrenal Scintigraphy as an Imaging Biomarker to Predict *KCNJ5* Mutation in Primary Aldosteronism Patients

**DOI:** 10.3389/fendo.2021.644927

**Published:** 2021-04-28

**Authors:** Ching-Chu Lu, Ruoh-Fang Yen, Kang-Yung Peng, Jei-Yie Huang, Kwan-Dun Wu, Jeff S. Chueh, Wan-Yu Lin

**Affiliations:** ^1^ Department of Nuclear Medicine, National Taiwan University Hospital, Taipei, Taiwan; ^2^ Institute of Epidemiology and Preventive Medicine, College of Public Health, National Taiwan University, Taipei, Taiwan; ^3^ Division of Nephrology, Department of Internal Medicine, National Taiwan University Hospital, Taipei, Taiwan; ^4^ Glickman Urological and Kidney Institute, Cleveland Clinic Lerner College of Medicine, Cleveland Clinic, Cleveland, OH, United States; ^5^ Department of Public Health, College of Public Health, National Taiwan University, Taipei, Taiwan

**Keywords:** primary aldosteronism, NP-59 adrenal scintigraphy, *KCNJ5*, semiquantification, mutation prediction

## Abstract

**Purpose:**

Somatic *KCNJ5* mutation occurs in half of unilateral primary aldosteronism (PA) and is associated with more severe phenotype. Mutation status can only be identified by tissue sample from adrenalectomy. NP-59 adrenal scintigraphy is a noninvasive functional study for disease activity assessment. This study aimed to evaluate the predictive value of NP-59 adrenal scintigraphy in somatic *KCNJ5* mutation among PA patients who received adrenalectomy.

**Methods:**

Sixty-two PA patients who had NP-59 adrenal scintigraphy before adrenalectomy with available *KCNJ5* mutation status were included. Two semiquantitative parameters, adrenal to liver ratio (ALR) and lesion to contralateral ratio of bilateral adrenal glands (CON) derived from NP-59 adrenal scintigraphy, of mutated and wild-type patients were compared. Cutoff values calculated by receiver-operating characteristic (ROC) analysis were used as a predictor of *KCNJ5* mutation.

**Results:**

Twenty patients had *KCNJ5* mutation and 42 patients were wild type. Patients harboring *KCNJ5* mutation had both higher ALR and CON (p = 0.0031 and 0.0833, respectively) than wild-type patients. With ALR and CON cutoff of 2.10 and 1.95, the sensitivity and specificity to predict *KCNJ5* mutation were 85%, 57% and 45%, 93%, respectively. Among 20 patients with *KCNJ5* mutation, 16 showed G151R point mutation (*KCNJ5*- G151R) and 4 showed L168R point mutation (*KCNJ5*-L168R), which former one had significantly lower ALR (p=0.0471).

**Conclusion:**

PA patients harboring somatic *KCNJ5* mutation had significantly higher NP-59 uptake regarding to ALR and CON than those without mutation. APAs with *KCNJ5-*L168R point mutation showed significantly higher ALR than those with *KCNJ5*-G151R point mutation.

## Introduction

Primary aldosteronism (PA) is the most common cause of secondary hypertension which is characterized by overproduction of aldosterone, leading to hypertension and sometimes hypokalemia (1). There are three concerns making PA an important issue. First, the prevalence is underestimated. Due to highly heterogeneity of each study, lack of uniform screening tests and cutoff values, it is difficult to conclude a definite prevalence. Roughly 10% is a commonly acceptable prevalence among hypertensive populations (1). However, prevalence of 30% has been reported, which may occur in severe hypertensive populations at tertiary referral center (2). Second, PA patients had significantly higher risk of cardiovascular events, diabetes and metabolic syndrome as compared with patients with essential hypertension (3). Third, PA is controllable and even curable through proper treatment, by means of mineralocorticoid antagonist for bilateral disease and adrenalectomy for unilateral disease (4). John W Funder has stated that PA is a public health issue on the basis of prior mentioned concerns and underlying great impact on medical care resources (5).

The major advance of understanding PA pathophysiology is the identification of *KCNJ5* mutation in aldosterone-producing adenomas (APAs) (6). Mutated *KCNJ5* leads to persistent cell depolarization turning out to aldosterone overproduction. Approximately 40% APAs harbored *KCNJ5* mutation, while eastern countries had much lower mutation rate than Asian countries. *KCNJ5* mutation is also associated with clinical phenotype. Younger age, higher plasma aldosterone, larger tumor, and female were more commonly seen with mutation (7). However, mutation status is only available by surgically resected specimen, which the mutation rate among PA patients will not be truly revealed.

NP-59 adrenal scintigraphy is a molecular imaging evaluating adrenal cortical function based on the activity of cholesterol uptake and transfer. It is able to correctly differentiate unilateral disease from bilateral disease and with excellent predictive value of postsurgical outcome (8, 9). Although adrenal venous sampling (AVS) is currently the gold standard for lateralization, NP-59 adrenal scintigraphy is an alternative method since AVS is technically dependent and invasive. Considering that more severe disease brings higher cholesterol demand to produce aldosterone, and *KCNJ5* mutation is associated with higher plasma aldosterone, we assume that NP-59 adrenal scintigraphy may be an imaging biomarker to predict *KCNJ5* mutation.

## Materials and Methods

### Patients

The study protocol was approved by the Institutional Review Board of National Taiwan University Hospital (approval No. 201002002R and 200912003R). Patients were retrospectively recruited from the Taiwan Primary Aldosteronism Investigation (TAIPAI) database with the following inclusion criteria: (1) clinically confirmed PA by either saline loading test or captopril test (a positive saline loading test is defined as post-test PAS higher than 10 ng/dl and a positive captopril test is defined as PAC suppression less than 30% of the baseline level concurrent with suppressed PRA or ARR > 35 ng/dl per ng/ml/h), (2) had NP-59 adrenal scintigraphy with single photon emission computed tomography (SPECT) and computed tomography (CT) before surgery, (3) underwent adrenalectomy within 1 year after NP-59 scintigraphy, and (4) available *KCNJ5* mutation status from surgical specimen. The only exclusion criteria was known malignancy with adrenal gland involvement. Adrenalectomy was determined by a successful non-stimulated AVS (which is defined as a selective index greater than 2) with a lateralization index than 2. Clinical and biochemical profiles were acquired at initial evaluation and 1 year after adrenalectomy.

### Protocol and Interpretation of NP-59 Adrenal Scintigraphy

Medications may alter NP-59 uptake such as glucocorticoids, diuretics, spironolactone, beta-blockers, alpha-blockers, and calcium channel blockers were postponed or switched to alternative medications (10). Oral dexamethasone suppression (8 mg daily) was carried out throughout the study for 8 days. One mCi NP-59 was slowly injected intravenously on the 4th day, and SPECT/CT was performed on the 96th hour after NP-59 injection. Patients were also given 1 ml of diluted Lugol’s solution daily to protect the thyroid from free ^131^I uptake.

Two semiquantitative parameters were used to evaluated adrenal cortical function as previously reported (9). Maximal count of the adrenal gland with lesion (defined as adrenal gland having adrenalectomy) divided by the mean count of the liver resulted in adrenal to liver ratio (ALR). Maximal count of adrenal gland with lesion to maximal count of contralateral adrenal gland is defined as CON.

### *KCNJ5* Sequencing

The specimens of APAs after adrenalectomy were collected and stored at −72°C until analysis. Genomic DNA were isolated from APAs using the QIAamp DNA mini kit (Qiagen, Hilden, Germany) according to the manufacturer’s instructions. The DNA regions containing the most frequently occurred point mutations of *KCNJ5*, p.Gly151Arg (G151R) and p.Leu168Arg (L168R), were amplified and sequenced using gene-specific primers (forward 5′-CTTCATTTGGTGGCTCATTGC-′3, reverse 5′-GGGACTTGATGAGCTTGGC-′3) as previously reported (11). The annealing temperature was 58°C. Direct sequencing of PCR products was performed using the BigDye^®^ Terminator v3.1 Cycle Sequencing Kit (Applied Biosystems, Foster City, USA) with a 3730 DNA Analyzer (Applied Biosystems, Foster City, USA). Sequences were analyzed using DNAStar Lasergene SeqMan Pro 7.1.0 software. Standard protocol of sequencing in TAIPAI followed that which had been previously reported (12).

### Statistical Analysis

Descriptive statistics were used for patients’ characteristics. Continues data were expressed as median with 25th percentile and 75th percentile. Mann-Whitney U test was used for data comparison. Difference of ALR and CON between mutated and wild-type patients were compared by Mann-Whitney U test. Receiver-operating characteristic (ROC) curves were plotted and areas under the curve (AUC) were calculated for ALR and CON. The sensitivity and specificity of ALR and CON to predict *KCNJ5* mutation were calculated according to the optimal cutoff value selected by Youden’s index. A p value of less than 0.05 was deemed statistically significant. All statistical analyses were performed using MedCalc Statistical Software version 17.9.2 (MedCalc Software bvba, Ostend, Belgium).

## Results

### Patient Characteristics

From October 2007 to October 2018, 64 patients were enrolled in this study. Two patients were excluded due to insufficient imaging information (one with missing data and the other with only SPECT imaging). Finally, 62 patients were enrolled for analysis, including 35 male (56%) and 27 female (44%), with a median age of 53 years (45–63). All patients received unilateral adrenalectomy and the median time interval between NP-59 adrenal scintigraphy and adrenalectomy was 134 days (75–249). The median ALR was 2.205 (1.69–2.98) and CON was 1.325 (1.14–1.83). The patients’ characteristics were listed in **Table 1**.

**Table 1:** Association between patients’ characteristics and *KCNJ5* mutation.

	Wild-type KCNJ5 (n = 42)	Mutated KCNJ5 (n = 20)	p
Age	54 (45–63)	53 (42–58)	0.2583
Gender	Male=24, Female=18	Male=11, Female=9	0.9164
Hypertension history (year)	5.5 (2–10)	4 (2.5–14)	0.8144
Size (cm)	1.55 (0.9–1.9)	1.55 (1.2–1.8)	0.5350
Preoperative antihypertensive medications	2 (2–3)	3 (1.5–3)	0.1158
Preoperative systolic BP (mm Hg)	156.5 (140–167)	142 (135.5–155)	0.0627
Preoperative diastolic BP (mm Hg)	95 (80–101)	86 (81–95)	0.1523
Preoperative PAC (ng/dl)	43.2 (26.6–56.44)	60.505 (43.465–79.525)	0.0130
Preoperative PRA (ng/ml/h)	0.275 (0.1–0.55)	0.34 (0.125–0.7)	0.1000
Preoperative ARR (ng/dl per ng/ml/h)	149.62 (60.04–577.88)	258.44 (79.97–578.875)	0.1944
Presence of hypokalemia before surgery	13 (31%)	12 (60%)	0.0546
Postoperative systolic BP (mm Hg)	139.5 (126–156)	125.5 (120.5–140.5)	0.0177
Postoperative diastolic BP (mm Hg)	83 (80–97)	78.5 (72–86)	0.0222
Postoperative PAC (ng/dl)	30.49 (23.635–54.11)	35.91 (27.15–42.475)	0.8351
Postoperative PRA (ng/ml/h)	0.99 (0.44–4)	3.98 (1.115–6.845)	0.0154
Postoperative ARR (ng/dl per ng/ml/h)	25.175 (10.545–71.72)	9.515 (5.94–31.875)	0.0557
Presence of hypokalemia after surgery	2 (5%)	1 (5%)	0.9682
ALR	2.005 (1.56–2.55)	2.815 (2.13–3.54)	0.0031
CON	1.69 (1.20–2.465)	1.315 (1.14–1.49)	0.0833

### Correlation Between Clinical/Biochemical Profiles and *KCNJ5* Mutation

Mutated patients had significantly higher preoperative plasma aldosterone concentration (PAC, 60.505 ng/dl [43.465–79.525] *versus* 43.2 ng/dl [26.6–56.44], p = 0.0130) and borderline higher portion of presence of hypokalemia before surgery (60% *versus* 13%, p = 0.0546). There were no differences regarding to age, gender, hypertension history, tumor size, preoperative antihypertensive medications, preoperative systolic blood pressure (BP), preoperative diastolic BP, preoperative plasma renin activity (PRA), and preoperative aldosterone to renin ratio (ARR).

There were significant differences regarding to postoperative clinical and biochemical profiles between mutated and wild-type patients. Significant lower postoperative systolic BP (125.5 mmHg [120.5–140.5] versus 139.5 mmHg [126–156], p = 0.02), diastolic BP (78.5 mmHg [72–86] *versus* 83 mmHg [80–97], p = 0.02), higher PRA (3.98 ng/ml/h [1.115–6.8456] *versus* 0.99 ng/ml/h [0.44–4], p = 0.02) and borderline lower ARR (9.515 ng/dl per ng/ml/h [5.94–31.875] *versus* 25.175 ng/dl per ng/ml/h [10.545–71.72], p = 0.06) were noted in mutated patients. Normalization of hypokalemia was seen in most patients except one mutated and two wild-type patients.

### Correlation Between NP-59 Adrenal Scintigraphy and *KCNJ5* Mutation

**Figure 1:**
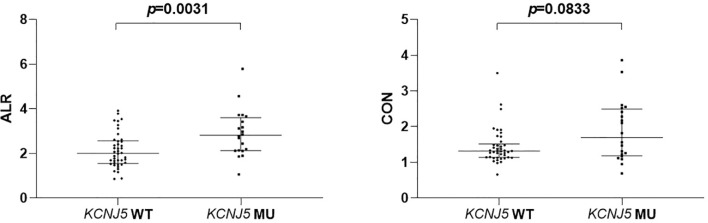
Comparison of semiquantitative parameters of NP-59 adrenal scintigraphy according to *KCNJ5* mutation status.

**Figure 2:**
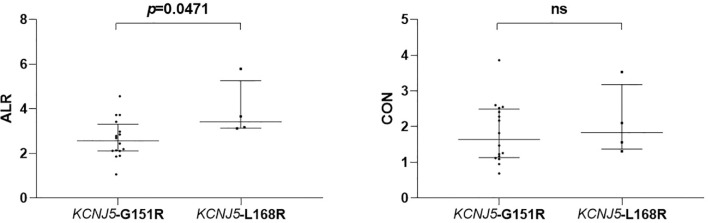
Comparison of semiquantitative parameters of NP-59 adrenal scintigraphy according to different point mutations of *KCNJ5*. ns, non-significant.

ALR were significantly higher in mutated patients than in wild-type patients (2.815 [2.13–3.54] *versus* 2.005 [1.56–2.55], p = 0.0031; **Figure 1**), while CON showed borderline higher in mutated patients (1.315 [1.14–1.49] *versus* 1.69 [1.20–2.456], p = 0.0833; **Figure 1**). ROC analysis showed that an ALR cutoff of 2.10 and a CON cutoff of 1.95 were the best values to predict *KCNJ5* mutation. By ALR cutoff of 2.10, the sensitivity, specificity, positive predictive value, negative predictive value, and accuracy were 85%, 57%, 49%, 89% and 66%, respectively. By CON cutoff of 1.95, the sensitivity, specificity, positive predictive value, negative predictive value, and accuracy were 45%, 93%, 75%, 78%, and 77%, respectively. Among 12 patients with both ALR greater than 2.1 and CON greater than 1.95, 10 harbored *KCNJ5* mutation (83%). Twenty-four of 27 patients (89%) having ALR less than 2.10 and CON less than 1.95 had wild-type *KCNJ5*. For patients with CON greater than 1.95, ALR was greater than 2.10 without exception. Representative SPECT/CT images of two patients with and without *KCNJ5* mutations were shown in **Figure 2**.

### G151R and L168R Point Mutations

**Figure 3:**
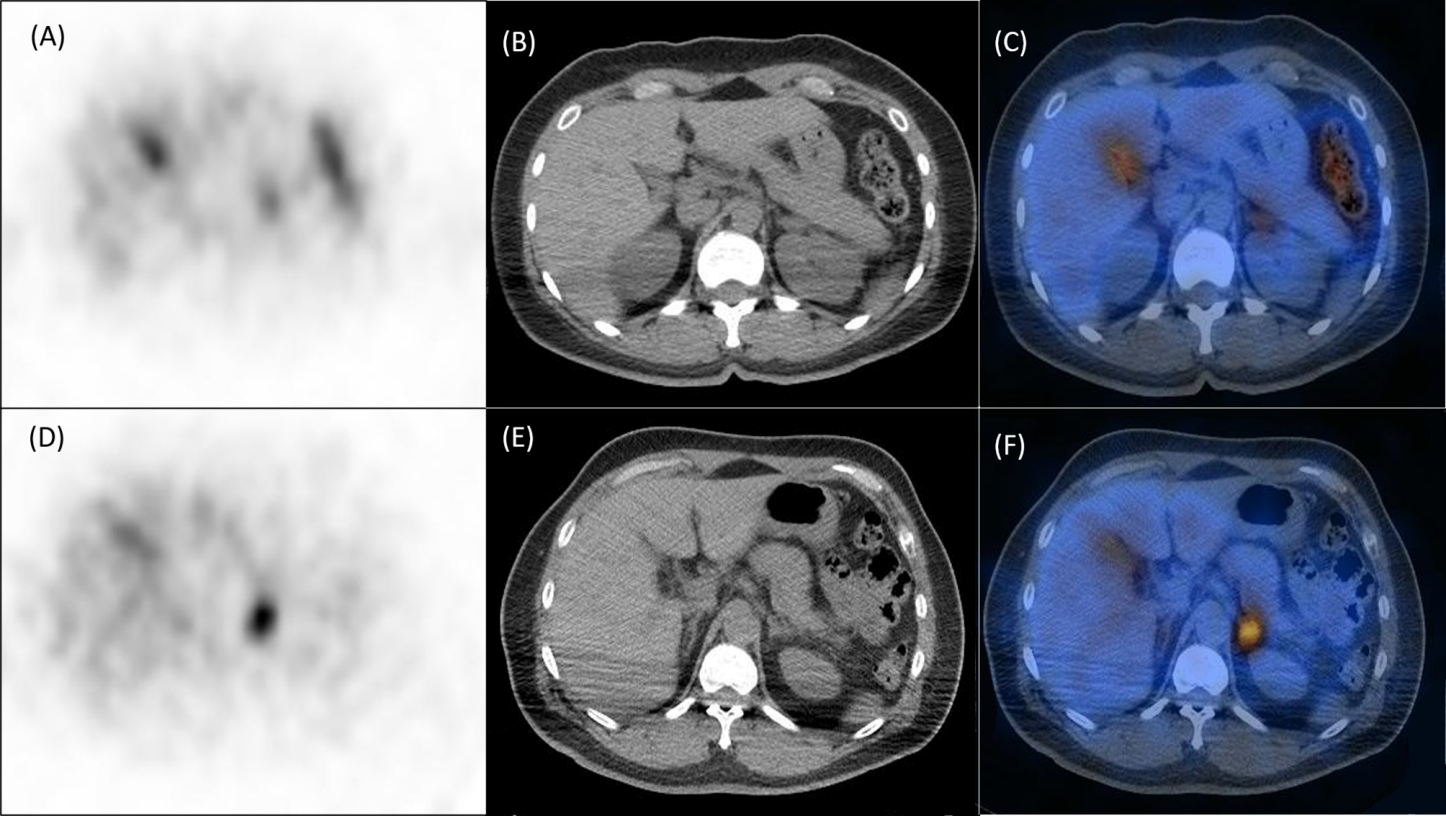
Representative NP-59 adrenal SPECT/CT and *KCNJ5* mutation status. Upper panel, findings of a 40-year-old female without *KCNJ5* mutation. SPECT **(A)**, CT **(B)** and fusion SPECT/CT **(C)** showed moderate NP-59 uptake at left adrenal gland with ALR of 2.62 and CON of 1.38. Lower panel, findings of a 54-year-old male with *KCNJ5* mutation. SPECT **(D)**, CT **(E)** and fusion SPECT/CT **(F)** showed significant NP-59 uptake at left adrenal gland with ALR of 3.72 and CON of 2.41.

Among 20 patients harboring *KCNJ5* mutation, 16 of them had G151R point mutation and 4 of them had L168R point mutation. There were no differences regarding to age, gender, hypertension history, tumor size, preoperative antihypertensive medications, preoperative systolic BP, preoperative diastolic BP, preoperative PAC, preoperative PRA and preoperative ARR. Postoperative clinical and biochemical profiles were similar except borderline lower PAC in patients with *KCNJ5-*L168R point mutation (20.05 ng/dl [12.24–32.15] *versus* 37.27 ng/dl [31.75–42.72], p = 0.07). ALR of *KCNJ5-*G151R was significantly lower than of *KCNJ5-*L168R point mutation (2.565 [2.115–3.20] *versus* 3.415 [3.145–4.725], p = 0.0471; **Figure 3** **)**. There was no difference between these point mutations regarding to CON (1.645 [1.145–2.465] *versus* 1.83 [1.435–2.815], p = 0.5536; **Figure 3**). ROC analysis showed that with cutoff of 2.98, ALR had best ability to differentiate *KCNJ5-*G151R from *KCNJ5-*L168R point mutation. The sensitivity, specificity, positive predictive value, negative predictive value, and accuracy were 75%, 100%, 100%, 50%, 80%, respectively. The patients’ characteristics were listed in **Table 2**.

**Table 2:** Characteristics of clinical and NP-59 adrenal scintigraphy regarding to different point mutation of *KCNJ5*.

	*KCNJ5*-G151R (n = 16)	*KCNJ5*-L168R (n = 4)	p
Age	53 (44–59)	48 (37–57)	0.5700
Gender	Male = 9, Female = 7	Male=2, Female=2	0.8676
Hypertension history (year)	6 (3–14)	3 (1.25–9.5)	0.3652
Size (cm)	1.5 (1.15–1.8)	2.05 (1.45–2.5)	0.1550
Preoperative antihypertensive medications	3 (1.5–3)	4 (2–5.5)	0.2624
Preoperative systolic BP (mm Hg)	141 (135.5–151)	156 (133.5–167)	0.2981
Preoperative diastolic BP (mm Hg)	86 (81–94)	92 (80–104.5)	0.5072
Preoperative PAC (ng/dl)	68.56 (43.465–79.525)	50.095 (37.5–103.195)	0.7768
Preoperative PRA (ng/ml/h)	0.39 (0.14–0.70)	0.12 (0.055–0.725)	0.3940
Preoperative ARR (ng/dl per ng/ml/h)	257.47 (79.97–416.565)	815.665 (292.2–2030.715)	0.3445
Presence of hypokalemia before surgery	9 (56%)	2 (50%)	0.8802
Postoperative systolic BP (mm Hg)	124.5 (119–140.5)	123.5 (115.5–126.5)	0.5082
Postoperative diastolic BP (mmHg)	80.5 (72–87)	76.5 (72–79)	0.3937
Postoperative PAC (ng/dl)	37.27 (31.75–42.72)	20.05 (12.24–32.15)	0.0725
Postoperative PRA (ng/ml/h)	3.98 (1.115–6.59)	4.055 (1.035–15.5)	0.8501
Postoperative ARR (ng/dl per ng/ml/h)	9.515 (6.33–32.025)	15.255 (1.04–31.875)	0.4497
Presence of hypokalemia after surgery	0	0	N/A
ALR	2.565 (2.115–3.20)	3.415 (3.145–4.725)	0.0471
CON	1.645 (1.145–2.465)	1.83 (1.435–2.815)	0.5536

## Discussion

In the present study, we found significant correlation between NP-59 uptake and *KCNJ5* mutation. Adrenal glands with tumor harboring *KCNJ5* mutation had higher NP-59 uptake than contralateral adrenal glands both by direct comparison (CON) and corrected by liver background (ALR). Furthermore, tumors with *KCNJ5*-G151R point mutation had significantly higher ALR than those with *KCNJ5*-L168R point mutation. To our knowledge, this is the first study analyzing SPECT/CT imaging to predict genetic mutation.

Five decades have passed since the first adrenal cortical imaging agent was developed (13). Basic concept is that cholesterol is the key component of hormones released from adrenal cortex, and radiolabeled cholesterol should be able to lead the way to imaging the factory. The original compound was ^125^I-19-iodochelesterol (14, 15) followed by first-in-human study by Beierwaltes et al. (16). Subsequent studies using ^131^I-19-iodochelesterol were applied to variety of adrenal diseases such as Cushing’s syndrome and primary aldosteronism (17–19). A chemical impurity ^131^I-6β-iodomethyl-19-norchelesterol (NP-59) was found during the synthesis of 19-iodochelesterol which showed three to five times higher adrenal uptake (20).

Criticism to NP-59 adrenal scintigraphy in PA lateralization also arose with long history, mainly based on limited imaging resolution resulting in poor ability to detect small APAs (21). Guideline by expert consensus has addressed that NP-59 adrenal scintigraphy has no role in subtype evaluation of PA (22). Great effort has been put on improving imaging quality by SPECT to illustrate adrenal lesions more clearly (23–25). We have previously reported the most comprehensive study using SPECT/CT to differentiate APA from idiopathic bilateral adrenal hyperplasia with sensitivity and specificity of 82% and 67%, respectively (8). Semiquantitative analysis could be easily performed from SPECT/CT imaging. We furthermore applied two semiquantitative parameters, ALR and CON, to predict postsurgical outcome which found that ALR and CON greater than the cutoff were significantly correlated with improvement of postsurgical outcome (9). In Taiwan mini-frontier of primary aldosteronism we suggested that NP-59 adrenal scintigraphy is an alternative method for lateralization when AVS is not available based on the above mentioned evidence (26).

Recognition of *KCNJ5* mutation in APAs by Choi et al. in 2011 is the most tremendous progression to understand the pathophysiology of PA. *KCNJ5* mutation altered the selectivity of encoding potassium channel which lead to cell membrane depolarization, influx of calcium ion and subsequent aldosterone production (6). *KCNJ5* is the most frequent genetic mutations in APAs with overall prevalence of 43%, ranging from 12% to 80% and it is widely studied for the correlation with phenotype, mainly in female, with younger age, larger tumor and higher PAC (7). Moreover, *KCNJ5* mutations were associated with postsurgical outcome. Kitamoto et al. found that patients with *KCNJ5* mutation had higher rate of hypertension resolution and decreased left ventricular hypertrophy after adrenalectomy (27). Vilela et al. also represented that *KCNJ5* mutation is the only independent predictor of hypertension remission (28). Change et al. recently demonstrated that mutation carriers had higher greater decrease in left ventricular mass index (LVMI) and inappropriately LVMI in a prospective cohort (29).

The importance of *KCNJ5* mutation arises from its high prevalence, phenotype association and postsurgical outcome correlation. However, *KCNJ5* mutation is only available with surgical specimen. Therefore, we aimed to utilize NP-59 adrenal scintigraphy as an imaging biomarker to predict *KCNJ5* mutation. In fact, it is common to use nuclear medicine imaging to predict disease biomarkers since it is noninvasive and easily to manipulate. Radiomics extracts quantitative imaging data and associates these features to relevant clinical profiles. Several studies have addressed the correlation between mutations and semiquantitative parameters of nuclear medicine imaging and the most relevant example is to predict *EGFR* mutation in lung cancer by 18F-fluorodeoxyglucose (FDG) positron emission tomography (PET). Maximal standard uptake value (SUVmax), which is the most commonly used semiquantitative parameter in PET, is associated with non-small cell lung cancer (NSCLC) with *EGFR* mutation and ALK positivity showing higher value (30). Radiomic features and conventional parameters (metabolic tumor volume and SUVmax) were proved to predictive of *EGFR* mutation in NSCLC (31). SUVmax is also shown to be correlated with *KRAS* mutation in colorectal cancer, *BRAF^V600E^* mutation in thyroid cancer and HER2 expression in gastric cancer (32–34).

In the present study we found that APA with *KCNJ5* mutation expressed significantly higher NP-59 uptake than those without *KCNJ5* mutation, in terms of ALR and CON. This correlation could be explained by the straightforward mechanism. Mutated channels alter the permeability of sodium which lead to depolarization of cell membrane and subsequent autonomous aldosterone production. Aldosterone production requires cholesterol as synthesis material and radiolabeled cholesterol, NP-59, is delivered to the overproduction side of adrenal gland. Mutated APAs produce more aldosterone than wild-type APAs which lead to more NP-59 uptake. In our cohort this genetic difference reflects both on the level of phenotype, preoperative PAC, and of molecular imaging, NP-59 adrenal scintigraphy.

AVS is currently the gold standard for lateralization and some studies have addressed the impact of *KCNJ5* mutation on lateralization index (LI). In 170 PA patients Seccia et al. evaluated 40 of them with selective index of AVS greater than 2.0 at both side, and found that LI was significantly higher in APA with *KCNJ5* mutations those without mutations (p = 0.02, detail value of LI not specified in the study) (35). Zheng et al. analyzed a larger Chinese cohort with 162 PA patients which revealed borderline higher LI in APA with *KCNJ5* mutations than those without mutations (10.9 [7.5–22.7] *versus* 6.4 [3.0–10.6], p = 0.053) (36). In contrast, Oßwald et al. found 19 PA patients harboring *KCNJ5* mutation had similar LI compared to 32 patients without *KCNJ5* mutation (20.5, interquartile range 30.3 *versus* 16.0, interquartile range 41.9) (37). The conflict and nonreplicated results not only attract attention of necessity of larger study, but also face up the contentious issues of AVS such as unstandardized protocol, different interpretation criteria and variable failed rate. NP-59 adrenal scintigraphy is an ancient nuclear medicine study with well-established protocol and sufficient evidence for both interpretation, lateralization and prognosis. Considering noninvasive nature and beneficial both for lateralization and mutation prediction, it is reasonable to have NP-59 adrenal scintigraphy before further treatment.

It is important to conduct the correlation between genotype and imaging findings into clinical practice, mainly referring to treatment. Predicting *EGFR* mutation by SUVmax of PET could identify proper candidate who may be beneficial from target therapy. In the present study we expect to use NP-59 adrenal scintigraphy selecting patients who may beneficial from medical treatment other than mineralocorticoid receptor antagonist (MRA). MRA is the drug of choice for bilateral disease as well as for patients who have contraindications to surgery or are not willing to receive surgery (which is common in Eastern countries) (22). The first line MRA is spironolactone acting by direct antagonizing the receptors which effectively lowers PAC. Side effects come along with its affinity to androgen receptor leading to gynecomastia and erectile dysfunction in male and to progesterone receptor leading to menstrual irregularity in female (38). In the SPARTACUS trial 57% of the patients developed these side effects from spironolactone compared with 1% of the adrenaletomy patients. This dose-dependent side effect has indeed limited the clinical application (39). Eplerenone is an alternative MRA when spironolactone is not tolerated or optimal BP is not achieved (22). It is selective for mineralocorticoid receptor without significant interaction with androgen or progesterone receptor and therefore it has much lower side effect compared to spironolactone (40). However, Eplerenone has less affinity to mineralocorticoid receptor and is only approved for PA use in Japan and USA, not in Europe, Australia and Taiwan (41, 42). Moreover, the price of eplerenone is 26 times compared to spironolactone in Taiwan. Epithelial sodium channel antagonists, such as amiloride, are the second-line choice for medical treatment of PA. It works as potassium-sparing diuretic which improves hypertension and hypokalemia. Although less effective than spironolactone, amiloride is generally well-tolerated due to lack of androgenic effect (43, 44). Calcium channel blockers (CCBs) could be a choice for PA treatment with variable mechanisms and effects. Dihydropyridine CCBs reduced BP by competing aldosterone binding to mineralocorticoid receptor (45). However, the concentration of most dihydropyridine CCBs is not able to block mineralocorticoid receptor at regular doses in treating hypertension (46). Several studies have been published to illustrate the pharmacological effects of above mentioned alternative medications regarding to *KCNJ5* mutation. Tauber et al. reported that amiloride and its analog EIPA blocked with Na^+^-permeable mutated *KCNJ5* cells with L168R point mutation. Although nondihydropyridine CCBs have no effect on mineralocorticoid receptor, verapamil and diltiazem showed potent inhibition of *KCNJ5-*L168R cells (47). Physicians are not able to know the effects of these alternative medications before prescription. In our cohort, patients harboring *KCNJ5*-L168R mutation had significantly higher ALR compared to those with *KCNJ5*-G151R mutation. It is of great novelty that amiloride and verapamil/diltiazem may work on patients with stronger potency and less side effects by the mutation status provided by NP-59 adrenal scintigraphy.

Recently macrolide antibiotics is proved to inhibit HEK293T cells transfected with mutated *KCNJ5* but not to wild-type cells without antibiotic activity (48). Thereafter Caroccia et al. proceeded the study which demonstrated that clarithromycin lowered CYP11B2 gene expression and aldosterone secretion in CD56+ cells ex vivo from *KCNJ5* mutated APAs, but not in CD56+ cells from wild-type APAs (49). NP-59 adrenal scintigraphy provides noninvasive method to predict *KCNJ5* mutation and may be able to identify patients beneficial to macrolide treatment. For patients intolerable to the dose-dependent side effect of spironolactone who may harboring *KCNJ5* mutation predicted by NP-59 adrenal scintigraphy, macrolide could be a choice to treatment regimen in the future, and the key point is to identify *KCNJ5* mutation in advance.

There are some limitations in our study. First, the retrospective, single-center design with relatively small sample size may lead to selection bias. Second, the patients in the present study were all Taiwanese. There is significant difference among races regarding to *KCNJ5* mutation rate. Higher *KCNJ5* mutation rate was noted in Eastern Asians (70%) compared to Caucasians (38%) (50). Validation of our results to general population requires more comprehensive survey. Third, other gene mutations such as *ATP1A1*, *ATP2B3*, *CACNA1D*, and *CTNNB1* account for a certain portion of somatic mutations in APA (51). Although these mutations were not found in the present cohort, it is not negligible and further study should be conducted.

In conclusion, our study suggested that semiqualitative parameters from NP-59 adrenal scintigraphy could predict *KCNJ5* mutations in PA patients. APAs harboring *KCNJ5* mutations had significantly higher ALR and borderline higher CON than those without *KCNJ5* mutations. Furthermore, APAs with *KCNJ5*-L168R mutation had significantly higher ALR than those with *KCNJ5*-G151R point mutation. Precision medicine, which individualized treatment based on distinct signature of each patient, is the trend of disease management. Our study is a proof-of-concept and the first study applying SPECT to predict mutation status which may be utilized in treatment plan of PA.

## Data Availability Statement

The data sets presented in this study can be found in online repositories. The names of the repository/repositories and accession number(s) can be found in the article/supplementary material.

## Ethics Statement

The studies involving human participants were reviewed and approved by the institutional review board of National Taiwan University Hospital. The patients/participants provided their written informed consent to participate in this study.

## Author Contributions

C-CL: data collection, imaging analysis, and manuscript writing. R-FY: imaging analysis. K-YP: genetic analysis. J-YH: statistical consult. K-DW: data collection. JC: data collection. W-YL: study design and correspondence. All authors contributed to the article and approved the submitted version.

## Funding

The study is partly supported by the grant provided from National Taiwan University Hospital (110-S5009).

## Conflict of Interest

The authors declare that the research was conducted in the absence of any commercial or financial relationships that could be construed as a potential conflict of interest.
